# The antitumor immune response in HER-2 positive, metastatic breast cancer patients

**DOI:** 10.1186/1479-5876-3-13

**Published:** 2005-03-22

**Authors:** Zorica D Juranic, Zora Neskovic-Konstantinovic, Tatjana P Stanojkovic, Zeljko Zizak, Tatjana Srdic, Nevenka Stanojevic-Bakic, Dusanka Milosevic, Danica Jovanovic

**Affiliations:** 1 Department of Experimental Oncology, Institute for Oncology and Radiology of Serbia, Pasterova 14 Belgrade, Serbia and Montenegro; 2 Department of Medical Oncology, Institute for Oncology and Radiology of Serbia, Pasterova 14 Belgrade, Serbia and Montenegro; 3 Department of Pathology, Institute for Oncology and Radiology of Serbia, Pasterova 14 Belgrade, Serbia and Montenegro

## Abstract

The aim of this study was to determine the basis for anti-tumor immune reactivity observed in patients with human epidermal growth factor receptor-2 (HER-2) (3+) breast carcinoma using an *in vitro *model in which the role of the HER-2-specific monoclonal antibody Herceptin was also investigated. Patients with metastatic breast cancer who had their primary tumor resected were included in this study. Peripheral blood mononuclear cell (PBMC)-dependent cytotoxicity in the presence or absence of Herceptin were assessed using the survival of target breast adenocarcinoma MDA-MB-361 cells as a parameter in a (3-(4,5-dimethyl-2-thiazolyl)-2,5-diphenyl-2H-tetrazolium bromide (MTT) test. We observed a significant increase in PBMC-dependent cytotoxicity when autologous serum was introduced in the assay. Furthermore, the addition of Herceptin significantly increases their cytotoxicity. These data suggest that autologous serum constitutively contains factors that might affect PBMC-dependent cytotoxic activity against HER-2 positive cancer cells.

## Introduction

In order to improve the unsatisfying results of current standard approaches to breast cancer treatment, therapeutic strategies tailored to individual patients' tumor phenotype based on better understanding of breast cancer biology should be considered. For instance, the human epidermal growth factor receptor-2 (HER-2/ErbB-2) is over-expressed in 25–30% of breast and ovarian cancers and has been broadly utilized as a target for passive immunotherapeutical interventions [1] in particular with the humanized anti-HER-2 monoclonal antibody trastuzumab (Herceptin) [2]. The mechanisms of anti-tumor activity of Trastuzumab are complex and still not fully understood. Trastuzumab induces rapid disappearence of HER-2 from the cell surface, thereby reducing heterodimer formation critical for the accumulation of the cyclin-dependent kinase inhibitor p27 resulting in cell cycle arrest [3]. It also appears that Trastuzumab suppresses VEGF expression, inhibits heregulin-mediated angiogenesis both *in vitro *and *in vivo*, reverses cytokine resistance, and restores E-cadherin expression [4]. Trastuzumab also inhibits constitutive HER-2 cleavage/shedding by metalloproteases and, consequently, the generation of phosphorylated p95 [3,5,6]. Since Trastuzumab contains a human immunoglobulin G1 (IgG1) Fc region, it is possible that the antibody may participate in complement mediated cytotoxicity (CMC) and/or antibody dependent cell-mediated cytotoxicity (ADCC). However, Trastuzumab cytotoxicity by CMC could not be documented experimentally [7], possibly because of the presence of membrane-bound complement regulatory proteins (mCRP) such as decay accelerating factor (DAF; CD55), membrane cofactor protein (MCP; CD46), or protectin (CD59) on the surface of breast carcinoma cells. However, its cytotoxicity through ADCC has been confirmed [5,6,8]. Herceptin-dependent cell-mediated cytotoxicity action could depend upon activation of FcγRI (CD64) in monocytes and dendritic cells and/or activation of FcγRIII, (CD16) in monocytes/macrofages and natural killer cells, which may release cytotoxic granules such as perforin and granzymes to destroy target tumor cells.

Suppression of Herceptin cytotoxicity could be mediated through FcγRII (CD32), constitutively expressed on monocytes, B cells, platelets, dendritic cells, eosinofils, basophiles and neutrophils. It is observed that Trastuzumab is particularly effective in patients with strong (immunohistochemistry score, IHC score = +3) overexpression of the HER-2 receptor, or medium over-expression (IHC score +2) [9]. However, passive immunotherapy of the breast cancer with Herceptin appears promising; it remains unclear why some patients with HER-2 positive tumors do not adequately respond to therapy. Therefore, the aim of this study was to identify *in vitro*, immune parameters, in the patients with breast carcinoma that may be relevant to anti-tumor immune modulation, and to estimate *in vitro *the capability of Herceptin to enhance anti-tumor activity in patients with breast cancer and normal controls.

## Patients, material and methods

Fourteen HER-2 positive advanced breast cancer patients were included in the study. All patients had histologically proven invasive breast cancer, diagnosed in the metastatic stage of the disease immediately before the inclusion (1 patient), or earlier in the operable clinical stages (13 patients). As the consequence of previous cytotoxic and/or endocrine treatment, most of the patients were postmenopausal at study entry. However, patients had been without any systemic endocrine or cytotoxic therapy at least one month before the serum sampling. All patients had visceral involvement, mostly including the liver metastases. All patients had HER-2 (3+) positive primary tumors according to immunohistochemical examination using DAKO HercepTest on paraffin embedded breast tumor specimens. At the time of inclusion, they were screened for randomization into the clinical study of chemotherapy with or without trastuzumab. Their characteristics are presented on Table 1.

**Table 1:** Patients' characteristics

			
Total No of BC patients			14
			
Age at study entry	range		36–69
(yrs)	median		52.5

			
Menstrual status	premenopausal		1
(No. of pts)	postmenopausal		13

			
Previous treatment	None		1
(No. of pts)	Surgery	radical (breast)	13
		biopsy only	1
		liver metastasectomy	2
	Irradiation – postoperative		4
	Systemic therapy	adjuvant	11
		adj./neo-adj. chemoth	9
		adj. endocrine therapy	5
		for metastatic disease	7

			
Min. time elapsed from discontinuation of previous treatments (days)			28

			
DFI (months)	range		0–144
	median		26

			
Histology type of primary tumor		IDC	8
(No. of pts)		ILC	2
		IC	4

			
Histology grade of primary tumor		1	0
(No. of pts)		2	8
		3	2
		unknown	4

			
SR status	Negative (ER neg. and PR neg.)		4
(No. of pts)	Positive (ER pos. and/or PR pos.)		10

			
HER-2 status	Positive (3+, ICH)		14
(No. of pts)			

			
Clinical stage of BC at study entry		Stage IV	14
			
	1^st ^disease relapse		7
	2^nd ^disease relapse		5
	3^rd ^disease relapse		2

			
Metastatic involvement		Visceral	14
(No. of pts)			
		liver involvement	11
		other organs involvement	8

BC = Breast cancer; DFI = Disease-free interval; IDC = Invasive ductal carcinoma;

ILC = Invasive lobular carcinoma; IC = Invasive carcinoma of unknown histology type;

SR = Steroid receptors; ER = Estrogen receptor; PR = Progesterone receptor;

HER-2 = Human epidermal growth factor receptor type 2.

The study was approved by Institutional Ethics Committee. All patients signed the written Informed Consent.

Twenty healthy donors, age range: 20–55 years, served as controls. Peripheral blood mononuclear cells (PBMC) cytotoxicity and PBMC and Herceptin-dependent cytotoxicity were assessed indirectly, through determination of target, HER-2 positive (3+), breast adenocarcinoma MDA-MB-361 cell survival, by 3-(4,5-dimethyl-2-thiazolyl)-2,5-diphenyl-2H-tetrazolium bromide) MTT test [10]. Twenty thousand target MDA-MB-361 cells (T) were mixed with effectors PBMC (E), at effectors to target (E:T) ratios of 0:1 and 5:1 for 48 h, in RPMI 1640 medium with 10% of control serum (pool of healthy sera), or these target cells were incubated in RPMI 1640 medium with 10% autologous serum and PBMC at E:T ratios: 0:1, 1.25:1, 2.5:1 and 5:1 with or without 21 μg/ml of Herceptin. After 48 h 20 μl of MTT solution (5 mg / ml phosphate buffered saline) were added and 4 h later 100 μl of 10% sodium dodecyl sulfate were added. The absorbance at 570nm of MTT stained wells were read at 570 nm 24 h latter.

To get the cell survival, the absorbance at 570 nm of MTT stained target cells (A) incubated :

• in nutrient medium with autologuous serum (Na),

• or in nutrient medium with autologuous serum plus efector cells (Na+E),

• or in nutrient medium with control serum (Nc),

• or in nutrient medium with control serum plus efector cells (Nc+E),

was always devided with the absorbance of control cells (Ac) i.e., target cells incubated in nutrient medium with control serum (Nc)

S(%) = A/Ac

It was implied that absorbance of corresponding blank AB was always substracted from A of samples, or from A of controls. Therefore final equation was:

S (%) = (A (_T+Na+E_)-A_(Na+E)_) / (A_(T+Nc) _-A_Nc_)

Percent of CD16^+ ^cells was determined by direct immunofluorescence assay using the anti-CD16 FITC monoclonal antibodies according to manufacturer's instructions (Becton Dickinson, San Jose, Ca, USA). Data collection and analysis were done on FacsCalibur™ flow cytometer (BDIS), using the CellQuest software (BDIS).

Statistical analysis was done using two-tailed Student's *t *test with a minima threshold for significance of *P *< 0.05).

## Results

**Figure 1:**
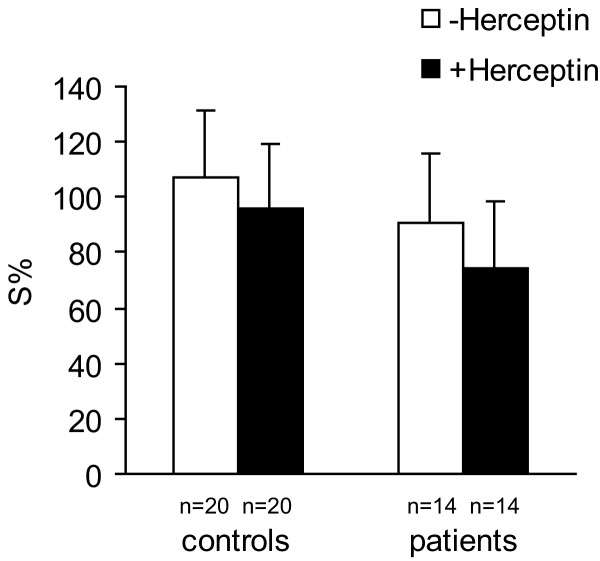
The mean values of the survival (S%) of adenocarcinoma MDA-MB-361 cells, grown for 48 h in nutrient medium with 10% serum from control healthy people, or from breast carcinoma patients with, or without Herceptin (21 μg/ml).

Serum isolated from healthy controls did not alter MDA-MB-361 cell growth compared with pooled serum. Sera from patients did not significantly suppressed the growth of target tumor cells (P = 0,05006) in comparison to their growth in pooled serum The survival of target cells grown in nutrient medium with serum of control healthy people or with serum of patients in the presence of 21 μg/ml of Herceptin was also not significantly affected (Figure 1).

**Figure 2:**
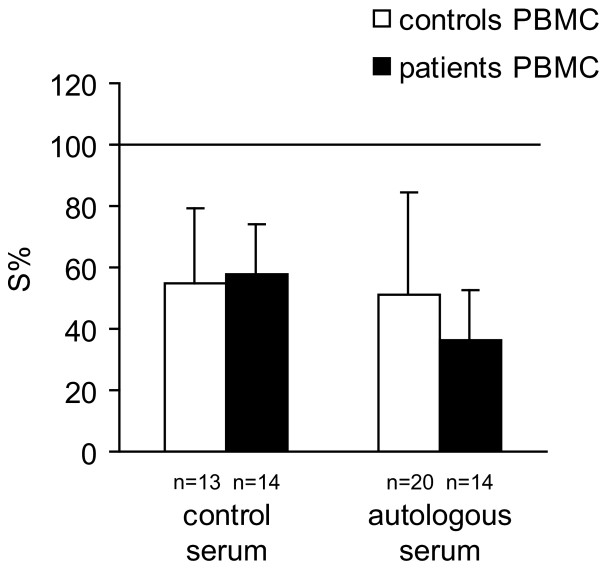
The mean values of survival (S%) of MDA-MB-361 cells co-cultured for 48 h, with effectors, PBMC isolated from control healthy people and from breast carcinoma patients, in nutrient medium with 10% of control, or autologous serum. E:T ratio was 5:1.

**Figure 3:**
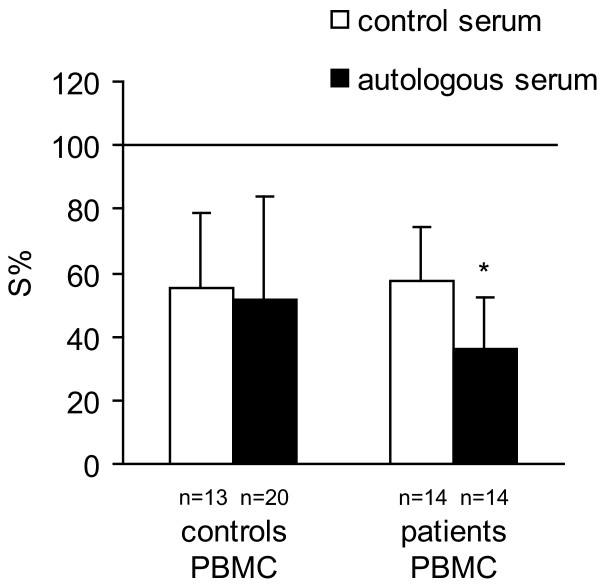
The mean values of the survival (S%) of MDA-MB-361 cells co-cultured for 48 h, with effectors PBMC, isolated from control healthy people and from the breast carcinoma patients, in nutrient medium with 10% of control, or autologous serum. E:T ratio was 5:1 (*P < 0.003).

PBMC from healthy donors manifested a non-significant trend toward higher cytotoxicity compared to PBMC from cancer patients when control pooled serum was used (as seen on Figure 2). Upon replacement of control serum with autologous serum, the cytotoxic activity of patients' PBMC was higher but not significantly (*P *= 0.09) than that of controls PBMC (Figure 2). Similarly, patients' PBMC activity was statistically enhanced (*P *< 0.003) by the presence of autologous serum compared to their action in pooled control serum (Figure 3). These data suggest that autologous serum of patients with breast cancer may contain a soluble facilitator of PBMC-mediated cytotoxicity, which is specific for each individual patient.

This anti-tumor humoral immunity present in autologous serum, which contributed to the enhancement (≥ 30%) of PBMC cytotoxicity, was seen in 5/14 HER-2 positive breast carcinoma patients, and in 4/13 healthy control subjects Therefore it seems that this is not a breast cancer patient specific phenomenon.

**Figure 4:**
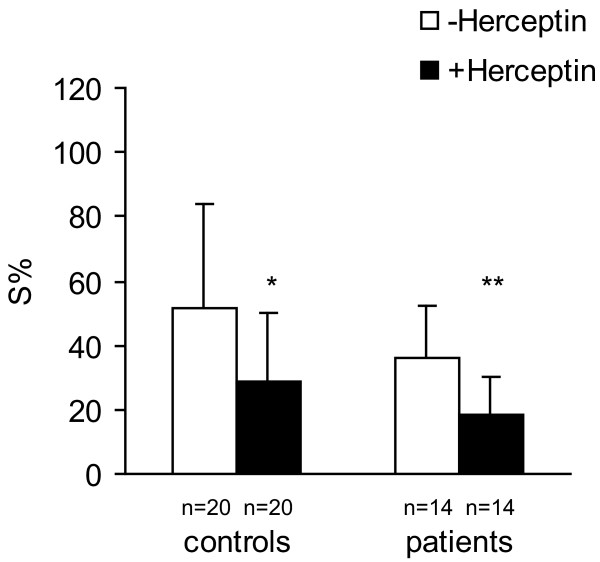
Herceptin in the concentration of 21 μg/ml in the incubation mixture of PBMC (from control healthy people and from breast carcinoma patients) and target cells decreased MDA-MB-361 cell survival (S%), in medium with 10% of autologous serum. E:T ratio was 5:1 (*P < 0.02, **P < 0.004).

The addition of Herceptin to PBMC from controls and from patients with breast cancer, significantly enhanced suppression of target cell survival at the E:T ratio 5:1 (*P *< 0.02 and *P *< 0.004 respectively) (Figure 4). This means that patients PBMC from breast cancer patients have not loosed their ability to kill malignant cells through Herceptin.

There were no statistically significant differences between the percentage of CD16^+ ^cells in patients and controls.

The clinical significance of the level of Herceptin dependent cytotoxicity in some patients treated with Herceptin is not available at the moment.

## Discussion

Addition of targeted HER-2-specific passive immune therapy for the treatment of patients with breast cancer may complement conventional therapy [11]. However, some patients do not successfully respond to such treatment. As Herceptin is primarily an immunological agent the potential of Herceptin to enhance anti-cancer immune responses in *vitro *might guide the selection of patients for Herceptin therapy. Our data suggest that MDA-MB-361 cells, over-expressing HER-2 are not significantly susceptible to direct Herceptin anti-proliferative action. This can be due to the loss of tumor suppressor PTEN by MDA-MB-361 cells which plays a central role in the transmission of growth inhibitory signals induced by Herceptin [12].

In addition, it appears according to our observations that PBMC from patients with breast cancer have non-significantly lower cytotoxic potential against MDA-MB-361 cells compared to PBMC isolated from healthy donors. However, patients PBMC in the presence of autologous serum significantly (*P *< 0.003) enhanced their immune reactivity possibly because of spontaneous humoral HER-2 anti-tumor immunity. These findings are in accordance with Disis et al. [13] reporting the existence of humoral immunity in some patients with breast cancers. Unexpectedly, the anti-tumor humoral immunity, which leads to decreased malignant cell survival was also found in a minority of healthy people.

The significance of the existence of an effective anti-tumor immunity *in vitro *in some patients with breast carcinoma which is not effective in destroying tumors *in vivo *remains unclear. Possibly, some proteins may inhibit the lytic action of perforin and granzymes. One of such proteins could be calreticulin that exerts several inhibitory actions including the inhibition of C1q-dependent complement activation [14]. This protein, together with perforin, is present in the cytolytic granule of NK and cytotoxic T cells protecting them from self destruction by perforin [15]. It has been suggested that calreticulin stabilizes membranes and indirectly prevents polyperforin pore formation, therefore, inhibiting target cell susceptibility to effector cell killing [16,17]. The over-expression of serpins by some tumor cells could also inhibit granzim A or granzyme B lytic activity too [18,19]. Results obtained in this work point to the new experiments aiming to determine the utility of *in vitro *immunological tests for the prediction of patients response to immunotherapy; i.e. the relevance of different levels of calreticulin and/or serpins in target cells used *in vitro *and in cells from malignant tumor specimens in immunological tumor escape needs to be determined.

Absence of statistically significant difference between percentage of CD16^+ ^cells in patients and in controls suggest that ADCC mediated by NK cells is likely preserved in patients with breast carcinoma.

**Table 2:** Survival of target tumor MDA/MB-361 cells incubated with breast cancer patients PBMC in nutrient medium with 10% pooled serum (Nc) or with 10% of autologous serum (Na) without or with 21 μg/ml Herceptin (Na+H). Effectors (E) to target tumor cells (T) ratio was 5:1.

Patient	S _N _(%)	S _Na _(%)	S _Na+H _(%)	CD16 (%)
1	73	30	21	32.02
2	42	39	13	18.98
3	73	69	30	16.68
4	49	27	24	25.76
5	87	23	12	ND
6	69	62	49	ND
7	55	33	5	28.00
8	53	11	8	25.32
9	49	40	11	5.47
10	51	57	33	4.84
11	24	34	13	ND
12	70	32	10	ND
13	40	35	19	11.81
14	74	14	9	25.59

ND – non-determined

**Table 3:** Survival of target tumor MDA/MB-361 cells incubated with healthy controls PBMC in nutrient medium with 10% pooled serum (Nc) or with 10% of autologous serum (Na) without or with 21 μg/ml Herceptin (Na+H). Effectors (E) to target tumor cells (T) ratio was 5:1.

Control	S _Nc _(%)	S _Na _(%)	S _Na+H _(%)	CD16 (%)
1	ND	103	49	15.00
2	ND	52	24	14.20
3	ND	73	55	10.00
4	ND	92	35	35.45
5	ND	66	17	ND
6	ND	22	26	ND
7	ND	89	58	ND
8	105	85	85	ND
9	58	85	49	17.55
10	74	82	34	16.15
11	20	27	0	16.45
12	93	92	24	ND
13	34	0	0	8.98
14	40	26	26	12.48
15	40	30	21	9.49
16	29	25	26	4.62
17	46	25	22	9.49
18	53	8.8	7	32.19
19	74	29	13	13.2
20	47	16	10	34.58

ND – non-determined
